# Clinical characteristics of the severe acute respiratory syndrome coronavirus 2 omicron variant compared with the delta variant: a retrospective case-control study of 318 outpatients from a single sight institute in Japan

**DOI:** 10.7717/peerj.13762

**Published:** 2022-08-02

**Authors:** Keiko Suzuki, Takaya Ichikawa, Satoshi Suzuki, Yoko Tanino, Yasutaka Kakinoki

**Affiliations:** 1 Department of General Internal Medicine, Asahikawa City Hospital, Asahikawa, Hokkaido, Japan; 2 Department of Hematology, Asahikawa City Hospital, Asahikawa, Hokkaido, Japan; 3 Department of Respiratory Medicine, Asahikawa City Hospital, Asahikawa, Hokkaido, Japan

**Keywords:** COVID-19, Outpatients, Risk factors, Omicron, Clinical characteristics

## Abstract

**Background:**

Clinical characteristics, including laboratory parameters, of the severe acute respiratory syndrome coronavirus 2 (SARS-CoV-2) Omicron variant have been limited.

**Methods:**

This retrospective case-control study was conducted in a single hospital. Patients with coronavirus disease 2019 (COVID-19) who visited the Asahikawa City Hospital outpatient department as new patients and underwent blood tests were included in this study. We analyzed the data from January 2022 to April 2022 during the Omicron phase and from April 2021 to October 2021 during the Delta phase. Patients who were treated at other hospitals after visiting our hospital were excluded. All blood tests were performed before treatment for COVID-19 was initiated. Demographic information, laboratory data, and clinical courses were extracted from electronic medical records. We matched the two groups by age and comorbidities and compared their characteristics. We also analyzed factors associated with pneumonia in the Omicron phase.

**Results:**

A total of 151 Omicron patients and 167 delta patients were analyzed in this study. The mean age, rate of comorbidities, and vaccination were significantly higher in the Omicron group. The number of patients with pneumonia or those requiring oxygen, admissions, or both was significantly lower in the Omicron group. Lactate dehydrogenase (LDH), C-reactive protein (CRP), ferritin, aspartate aminotransferase (AST), and neutrophil-to-lymphocyte ratio (NLR) levels were significantly lower in the Omicron group. Compared with the mild symptom and pneumonia groups in the Omicron group, older age, higher body mass index (BMI), higher non-vaccination, higher LDH, and higher CRP levels were associated with the pneumonia group.

**Conclusion:**

The Omicron variant is associated with a reduction in hospitalization and the risk of pneumonia compared to the delta variant in a real-life clinical setting. In the Omicron variant, the risk of pneumonia is related to high-risk factors, laboratory data such as LDH and CRP levels, and no vaccination.

## Introduction

The Omicron variant is reportedly less pathogenic than the delta variant. Several studies have shown fewer hospital admissions, lower oxygen requirements, and lower mortality risk with the Omicron variant (Dyer, 2021, Maslo et al., 2021; Bouzid et al., 2022).

There are many reports concerning the risk factors and biomarkers associated with the worsening of coronavirus disease 2019 (COVID-19) in the Alpha and Delta variants. Vahey et al. (2021) reported that chronic hypoxemic respiratory failure with a requirement for oxygen supplementation, opioid use, metabolic syndrome, obesity, age ≥ 65 years, hypertension, arrhythmia, and male sex were significantly associated with the risk of hospitalization among patients with COVID-19. In addition, the levels of lactate dehydrogenase (LDH), D-dimer, C-reactive protein (CRP), ferritin, and neutrophil-to-lymphocyte ratio (NLR) at hospital admission are predictors of the prognosis for COVID-19 (Ayanian et al., 2020; Poudel et al., 2021; Li et al., 2020; Rahman et al., 2021; Kilercik et al., 2021). However, these studies were based on data from patients with the Delta and Alpha variants.

The characteristics of laboratory parameters in the Omicron variant remain unclear. Zhang et al. (2022) analyzed the data of hospitalized patients with the Omicron variant and showed that CRP and LDH were abnormal in 20.7% and 37.9% of the patients, respectively. Bouzid et al. (2022) showed that CRP and D-dimer levels are lower in the Omicron variant group than in the delta variant group in emergency departments. In this study, we analyzed COVID-19 outpatient data and compared the clinical characteristics of the Omicron and Delta variants. Furthermore, we divided the patients during the Omicron phase into mild symptom and pneumonia groups and determined which factors were associated with pneumonia.

**Figure 1:**
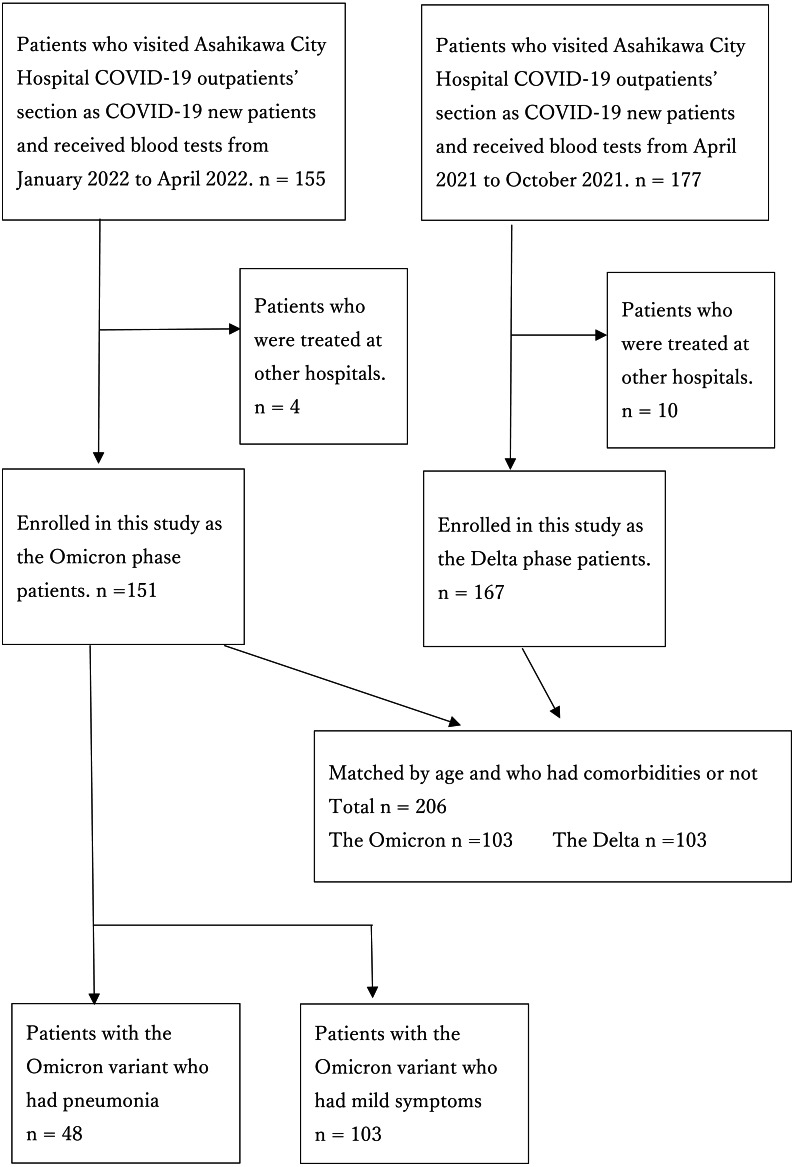
Flow chart of the participants.

## Materials & Methods

### Study design and participants

This retrospective case-control study was conducted in a single hospital. Patients newly diagnosed with COVID-19 who visited Asahikawa City Hospital as new patients and underwent blood tests were enrolled in the study. We divided them into two groups: the Delta group from April 2021 to October 2021 and the Omicron group from January 2022 to April 2022 (Fig. 1). All patients were diagnosed using polymerase chain reaction (PCR) testing for severe acute respiratory syndrome coronavirus 2 (SARS-CoV-2) genes, antigen quantification testing, or both from April 2021 to January 2022. According to the notification in February 2022 from the Ministry of Health, Labour, and Welfare of Japan, some symptomatic patients who lived in close contact with confirmed patients with COVID-19 were deemed positive with no testing. Our study included only patients over 15 years of age. Patients who had been treated at other hospitals were excluded. Furthermore, we divided the patients into two groups: those categorized as mild and those with pneumonia (Fig. 1). Multivariate logistic analysis was used to identify pneumonia-related factors in patients with the Omicron variant.

All demographic, clinical, and outcome data were retrospectively extracted from the records of the Asahikawa City Hospital. Demographic characteristics of the patients (age, sex, body mass index (BMI)); white blood cell count (WBC); neutrophil-lymphocyte ratio (NLR); lactate dehydrogenase (LDH), aspartate aminotransferase (AST), alanine aminotransferase (ALT), D-dimer, and ferritin levels, and comorbidities were recorded. All blood samples were collected before patients received treatment for COVID-19.

### Statistics

Continuous data were analyzed using the Mann–Whitney *U* test and presented as the mean (interquartile range). Fisher’s exact test or chi-square test was used for categorical variables, as appropriate.

Statistical significance was set at *P* < 0.05. Statistical analysis was performed using EZR (Saitama Medical Center, Jichi Medical University, Saitama, Japan), a 120 graphical user interface for R (The R Foundation for Statistical Computing, Vienna, 121 Austria) (Kanda, 2013).

We created matched Omicron and Delta groups by age and comorbidities using a caliper width of 0.2.

### Ethics

This study was approved by the ethics committee of Asahikawa City Hospital (No. 8, 2021) and was conducted in accordance with the ethical standards outlined in the Declaration of Helsinki (1983). An opt-out method was used to obtain patient consent.

## Results

### Characteristics

A total of 332 outpatients with COVID-19 who visited Asahikawa City Hospital and underwent blood tests were enrolled in the study. We excluded 14 patients treated in other hospitals after our hospital owing to missing clinical course data. The number of Delta phase patients was 167 from April 2021 to October 2021, and the number of Omicron phase patients was 151. All blood samples were collected in an outpatient setting.

Table 1 presents the characteristics of the 318 patients. The median patient age was significantly higher in the Omicron phase than in the Delta phase, 62 (44.0–78.5) years *versus* 48 (39–56) years (*p* < 0.001). The patient comorbidity rate is higher in the Omicron group (64.9%) than in the Delta group (42.5%) (*p* < 0.001). The proportion of vaccinated patients was significantly higher in the Omicron group than in the Delta group (82.9% *vs.* 11.4%, *p* < 0.001). Patients with pneumonia, admission rate, and oxygen requirements were significantly lower in the Omicron group (31.8% *vs.* 62.9%, *p* < 0.001), 40.4% compared in the Delta group 56.9% (*p* = 0.004), and 12.6% in the Omicron group compared to 24.0% in the Delta group (*p* = 0.010). No significant differences were found in sex, BMI, or smoking history between the two groups. Table S1 shows the types of oxygen therapy used by the patients. There were no patients who died in the Omicron phase, and one patient died in the Delta phase. None of the patients required intensive care unit therapy. In the Omicron group, higher WBC (5,440/µL [3,980–6,755] *vs.* 4,700/µL [3,845–5,685]/µL, *P* = 0.005), higher Platelet (Plt) (182*10^3^/µL [147–230*10^3^] *vs.*. 155*10^3^ /µL[131–196*10^3^], *P* < 0.001), lower LDH (200 U/L [173–220] *vs.* 238 U/L [195–305], *P* < 0.001), lower NLR (2.87 [1.82–4.35] *vs.*. 3.42 [2.23–5.33], *P* = 0.035), lower CRP (0.975 mg/dL [0.36–3.55] *vs.* 2.43 mg/dL [0.87–4.89], *P* = 0.001), lower ferritin (184.4 ng/mL [88.6–290] *vs.* 284 ng/mL [106–523], *P* = 0.004), lower AST (25.0 U/L [19.0–34.0] *vs.* 31.0 U/L [23.0–41.0], *P* < 0.001), lower ALT (21.0 U/L [14.0–35.0] *vs.* 24.0 U/L[17.0–43.0], *P* = 0.002) and higher D-dimer (0.6 µg/mL [0.3–1.2] *vs.* 0.5 µg/mL [0.2–1.0], *P* = 0.046) were found compared to the Delta group.

**Table 1:** Characteristics of 151 Omicron patients and 167 Delta patients.

	**Omicron (*n* = 151)**	**Delta (*n* = 167)**	***P***
**Demographics**			
Male (%)	85 (56.3%)	99 (59.3%)	0.649
Age (years), median (IQR)	62.0 (44.0–78.5)	48.0 (39.0–56.0)	<0.001*
BMI, median (IQR) (missing: 15.0%)	23.7 (21.4–27.0)	24.2 (21.7–27.3)	0.699
Smoking, yes (%) (missing: 33.0%)	45 (40.5%)	70 (43.5%)	0.708
SARS-CoV-2 vaccines, yes (%) (missing:3.8%)	116 (82.9%)	19 (11.4%)	<0.001*
Comorbidities (%)	98 (64.9%)	71 (42.5%)	<0.001*
Pneumonia, yes (%)	48 (31.8%)	105 (62.9%)	<0.001*
Admission, yes (%)	61 (40.4%)	95 (56.9%)	0.004*
Oxygen requirement (%)	19 (12.6%)	40 (24.0%)	0.010*
**Laboratory findings**			
WBC (/µL), mean (IQR)	5,440 (3,980–6,755)	4,700 (3,845–5,685)	0.005*
Plt (10^3^/µL)	182 (147–230)	155 (131–196)	<0.001*
Neutrophil-to-lymphocyte ratio, mean (IQR)	2.87 (1.82–4.35)	3.42 (2.23–5.33)	0.035*
AST (U/L), mean (IQR)	25.0 (19.0–34.0)	31.0 (23.0–41.0)	<0.001*
ALT (U/L), mean (IQR)	21.0 (14.0–35.0)	24.0 (17.0–43.0)	0.002*
LDH (U/L), mean (IQR)	200 (173–220)	238 (195–305)	<0.001*
Ferritin (ng/mL), mean (IQR) (missing: 14.1%)	184.4(88.6–290)	284 (106–523)	0.004*
D-dimer (µg/mL), mean (IQR) (missing: 13.2%)	0.6 (0.3–1.2)	0.5 (0.2–1.0)	0.046*
CRP (mg/dL), mean (IQR)	0.975 (0.36–3.55)	2.43 (0.87–4.89)	0.001*

**Notes.**

An asterisk (*) indicates *p* value smaller than 0.05 (*p* < 0.05).

Table 2 shows the data of the matched groups in terms of age and comorbidities. The proportion of vaccinated patients was significantly higher in the Omicron group (83.7% *vs.* 8.8%, *p* < 0.001). Patients who developed pneumonia, required oxygen, or required admission were significantly lower in the Omicron group, 25.2% *versus* 71.8% (*p* < 0.001), 32.0% *versus* 66.0% (*p* < 0.001), and 5.8% *versus* 29.1% (*p* < 0.001). No significant differences were found in sex, BMI, or smoking history between the two groups.

**Table 2:** The AUC (95% confidence interval [CI]), *P*-value, cutoff, sensitivity, and specificity of WBC, NLR, CRP, AST, and LDH in predicting the need for oxygen therapy.

	**Omicron (*n* = 103)**	**Delta (*n* = 103)**	***P***
**Demographics**			
Male (%)	56 (54.4%)	60 (58.3%)	0.674
Age (years), median (IQR)(matched)	50.0 (41.0–64.5)	49.0 (40.5–63.5)	0.990
BMI, median (IQR) (missing: 15.0%)	23.5 (21.4–28.2)	24.2 (22.0–28.1)	0.749
Smoking, yes (%) (missing: 12.1%)	35 (43.8%)	47 (46.5%)	0.764
SARS-CoV-2 vaccines, yes (%) (missing:3.8%)	82 (83.7%)	9 (8.8%)	<0.001*
Comorbidities (%) (matched)	54 (52.4%)	54 (52.4%)	1
Pneumonia, yes (%)	26 (25.2%)	74 (71.8%)	<0.001*
Admission, yes (%)	33 (32.0%)	68 (66.0%)	<0.001*
Oxygen requirement (%)	6 (5.8%)	30 (29.1%)	<0.001*
**Laboratory findings**			
WBC (/µL), mean (IQR)	5,610 (4,010–6,780)	4,510 (3,775–5,470)	0.004*
Plt (10^3^/µL)	193 (156–235)	150 (129–195)	0.034*
Neutrophil-to-lymphocyte ratio, mean (IQR)	2.96 (1.74–4.31)	3.48 (2.25–5.39)	0.035*
AST (U/L), mean (IQR)	18.5 (13.0–34.5)	31.0 (24.5–39.5)	<0.001*
ALT (U/L), mean (IQR)	23.0 (14.0–36.5)	24.0 (17.0–39.5)	0.117
LDH (U/L), mean (IQR)	197 (172–220)	250 (199–325)	<0.001*
Ferritin (ng/mL), mean (IQR) (missing: 14.1%)	169 (86–340)	272 (103–568)	0.028*
D-dimer (µg/mL), mean (IQR) (missing: 13.2%)	0.5 (0.2–1.1)	0.5 (0.3–1.1)	0.877
CRP (mg/dL), mean (IQR)	0.91 (0.31–3.11)	2.74 (1.25–5.63)	<0.001*

**Notes.**

An asterisk (*) indicates *p* value smaller than 0.05 (*p* < 0.05).

In the Omicron group, higher WBC (5,610/µL [4,010–6,780] *vs.* 4,510/µL [3,775–5,470]/µL, *P* = 0.004), higher Platelet (193*10^3^/µl [156–235*10^3^] *vs.* 150*10^3^/µl [129–195*10^3^], *P* < 0.001), lower LDH (197 U/L [172–220] *vs.* 250 U/L [199–325], *P* < 0.001), lower NLR (2.96 [1.74–4.31] *vs.* 3.48 [2.25–5.39], *P* = 0.035), lower CRP (0.91 mg/dL [0.31–3.11] *vs.* 2.74 mg/dL [1.25–5.63], *P* < 0.001), lower ferritin (169 ng/mL [86–340] *vs.* 272 ng/mL [103–568], *P* = 0.028), and lower AST (18.5 U/L [13.0–34.5] *vs.* 31.0 U/L [24.5–39.5], *P* < 0.001) were found compared to the Delta group. ALT and D-dimer levels did not differ between the groups.

In the Omicron phase, 103 patients were categorized into the mild group and 48 into the pneumonia group. Table 3 shows the data of 151 patients with the Omicron variant. The median patient age was significantly higher in the pneumonia group than in the mild group, 71.5 (56.8–84.0) years *versus* 56.0 (42.0–74.5) years (*p* = 0.001). The proportion of men was significantly higher in the pneumonia group (70.8% *vs.* 49.5%) (*p* = 0.015). The median BMI was higher in the pneumonia group, 25.3 (23.0–27.8) *vs.* 23.4 (20.8–26.3) (*p* = 0.029). There were no significant differences in smoking, vaccination, and comorbidities.

**Table 3:** Univariate analysis of the Omicron patients, compared with the mild group and the pneumonia group.

	**Mild *n* = 103**	**Pneumonia *n* = 48**	***P***	**Odds ratio**
**Demographics**				
Male (%)	51 (49.5%)	34 (70.8%)	0.015*	2.48 (1.19–5.15)
Age (years), median (IQR)	56.0 (42.0–74.5)	71.5 (56.8–84.0)	0.001*	1.03 (1.01–1.05)
BMI, median (IQR) (missing: 15.2%)	23.4 (20.8–26.3)	25.3 (23.0–27.8)	0.029*	1.08 (0.99–1.18)
Smoking, yes (%) (missing: 26.5%)	29 (38.2%)	16 (45.7%)	0.534	1.36 (0.61–3.07)
SARS-CoV-2 vaccines, yes (%)	84 (85.7%)	32 (76.2%)	0.221	0.53 (0.22–1.32)
Comorbidities (%)	62 (75.0%)	36 (60.2%)	0.099	1.98 (0.92–4.26)
**Laboratory findings**				
WBC (/µL), mean (IQR)	5,490 (4,290–6,770)	5,220 (3,570–6,598)	0.312	1.00 (1.00–1.00)
Plt (10^3^/µL)	185 (156–235)	167 (133–215)	0.027*	0.99 (0.99–1.00)
NLR, mean (IQR)	2.84 (1.69–4.30)	2.92 (2.15–4.56)	0.318	1.00 (0.98–1.01)
AST (U/L), mean (IQR)	23.0 (18.0–33.5)	31.0 (22.0–36.0)	0.006*	1.00 (0.99–1.02)
ALT (U/L), mean (IQR)	17.0 (13.0–32.5)	25.0 (15.7–35.3)	0.160	1.00 (0.99–1.02)
LDH (U/L), mean (IQR)	189 (170–207)	221 (199–254)	<0.001*	1.02 (1.01–1.03)
Ferritin (ng/mL), mean (IQR) (missing: 15.0%)	134 (71–240)	274 (172–578)	<0.001*	1.00 (1.00–1.00)
D-dimer (µg/mL), mean (IQR) (missing: 12.7%)	0.5 (0.3–1.1)	0.7 (0.5–1.5)	0.012*	1.11 (0.93–1.32)
CRP (mg/dL), mean (IQR)	0.72 (0.22–1.83)	3.13 (0.83–6.33)	<0.001*	1.33 (1.16–1.53)

**Notes.**

An asterisk (*) indicates *p* value smaller than 0.05 (*p* < 0.05).

In the pneumonia group, lower Plt (167*10^3^/µl [133–215*10^3^] *vs.* 185*10^3^/µl [156–235*10^3^], *P* = 0.027), higher LDH (221 U/L [199–254] *vs.* 189 U/L [170–207], *P* < 0.001), higher CRP (3.13 mg/dL [0.83–6.33] *vs.* 0.72 mg/dL [0.22–1.83], *P* < 0.001), higher ferritin (274 ng/mL [172–578] *vs.* 134 ng/mL [71–240], *P* < 0.001), higher D-dimer (0.7 µg/mL [0.5–1.5] *vs.* 0.5 µg/mL [0.3–1.1], *P* = 0.012) and higher AST (31.0 U/L [22.0–36.0] *vs.* 23.0 U/L [18.0–33.5], *P* = 0.006) were found compared to the mild group. The WBC, NLR and ALT levels did not differ between the groups. Multivariate logistic regression analysis was performed on the data. To address the problem of multicollinearity, we excluded the categories smoking, WBC, AST, and ALT. The adjusted odds ratio of age was 1.06 (1.01–1.11, *P* = 0.020), BMI was 1.19 (1.02–1.40, *P* = 0.030), SARS-CoV-2 vaccines was 0.12 (0.02–0.55, *P* = 0.006), LDH was 1.03 (1.01–1.05, *P* = 0.006) and CRP was 1.50 (1.19–1.89, *P* = 0.001) (Table 4). There were no significant differences in sex, comorbidities, Plt, NLR, D-dimer and Ferritin. The multivariate analysis showed that older age, higher BMI, more non-vaccination, higher LDH, and higher CRP levels were associated with the pneumonia group.

**Table 4:** Multivariate analysis of the Omicron patients, compared with the mild group and the pneumonia group.

	**Adjusted Odds Ratio**	***P***
**Demographics**		
Male (%)	1.74 (0.45–6.75)	0.424
Age (years)	1.06 (1.01–1.11)	0.020*
BMI	1.19 (1.02–1.40)	0.030*
SARS-CoV-2 vaccines, yes (%)	0.12 (0.02–0.55)	0.006*
Comorbidities (%)	0.55 (0.12–2.49)	0.443
**Laboratory findings**		
Plt (10^3^/µL)	1.00 (0.92–1.01)	0.622
NLR	1.00 (0.98–1.02)	0.825
LDH (U/L)	1.03 (1.01–1.05)	0.006*
D-dimer (µg/mL) (missing: 12.7%)	1.20 (0.92–1.56)	0.176
Ferritin (ng/mL) (missing: 15.0%)	1.00 (1.00–1.00)	0.938
CRP (mg/dL)	1.50 (1.19–1.89)	0.001*

**Notes.**

An asterisk (*) indicates *p* value smaller than 0.05 (*p* < 0.05).

## Discussion

According to our outpatient data, the percentage of patients with pneumonia, required oxygen, and required admission were significantly lower in the Omicron group than in the Delta group. Bouzid et al. (2022) analyzed the patients in emergency departments and showed that the Omicron variant had a lower rate of ICU admission, mechanical ventilation, and in-hospital mortality. Luliano et al. (2022) used data from the Center for Disease Control and Prevention in the United States and reported that the Omicron variant had a lower ICU admission and death rate than the Delta.

During the phase change, vaccination rates increased, and new medicines such as sotrovimab, nirmatrelvir/ritonavir, and molnupiravir have been available to treat patients with mild COVID-19. Although there is a possibility that the new medicine contributed to decreasing the severity in patients with the Omicron variant, the effects of these medicines on vaccinated patients are still unknown; therefore, further studies are needed.

Three vaccine doses are effective against the Omicron variant (Lauring et al., 2022). Lauring et al. analyzed data from hospitalized patients and showed that the severity of COVID-19 was lower in the Omicron group than in the Delta group, even among unvaccinated cases (Lauring et al., 2022). In addition, the data showed that patients with the Omicron variant had milder characteristics without the influence of the vaccines.

Concerning laboratory data, our study indicated that LDH, CRP, ferritin, AST, and NLR levels were significantly lower in the Omicron group than in the Delta group. These findings may be attributed to the milder characteristics of the Omicron variant. Only a few studies have been conducted on laboratory data in the Omicron phase. Zhang et al. (2022) analyzed the data of hospitalized Omicron patients and showed that CRP was abnormal in 20.7% and 37.9% of the patients, respectively. Bouzid et al. (2022) showed that CRP and D-dimer levels are lower in the Omicron group than in the Delta group in emergency departments.

In this study, compared with the mild symptom and pneumonia groups during the Omicron phase, older age, higher BMI, more non-vaccination, higher LDH, and higher CRP levels were associated with the pneumonia group. These findings are similar to those of previous studies on the Delta and Alpha variants. The US Food and Drug Administration published criteria for the use of monoclonal antibodies to treat or prevent SARS-CoV-2 in consideration of risk factors, including older age (≥ 65 years), obesity (BMI >25 kg/m^2^ in adults), pregnancy, and comorbidities such as chronic kidney disease, diabetes, and chronic lung disease (Drug Administration, 2021). Biomarkers for inflammation and coagulopathy can aid in identifying patients hospitalized with COVID-19 who are at risk of clinical deterioration (Ayanian et al., 2020). CRP levels in the early stage of COVID-19 have been positively correlated with the presence of lung lesions (Wang, 2020). Patients with higher CRP levels on admission were more likely to have severe complications of COVID-19 (Sadeghi-Haddad-Zavareh et al., 2021). LDH is an inflammatory marker that may serve as a common indicator of acute or severe tissue damage (Sepulveda, 2019). Higher LDH levels during hospitalization are associated with moderate-to-severe acute respiratory distress syndrome in patients with COVID-19 (Poggiali et al., 2020).

Regarding study limitations, the number of cases was small, and this study was performed retrospectively at a single hospital. The sample size was small, especially for the multivariate model; therefore, the two groups may not be comparable. Only some patients underwent blood testing during their visits based on the judgment of the physicians. Since all patients were ethnically Japanese, the results should be verified in other populations. Further studies are required to confirm these findings.

## Conclusions

The percentage of patients with pneumonia, who required oxygen, and who required admission was significantly lower in the Omicron group. In addition, LDH, CRP, ferritin, AST, and NLR levels were significantly lower in the Omicron group. Compared with the mild symptom and pneumonia groups during the Omicron phase, older age, higher BMI, more non-vaccination, higher LDH, and higher CRP levels were associated with the pneumonia group.

## Supplemental Information

10.7717/peerj.13762/supp-1 Table S1 The type of oxygen therapy used for the patients

10.7717/peerj.13762/supp-2 Table S2 STROBE checklists

10.7717/peerj.13762/supp-3 Table S3 Raw data of 318 patients

## Additional Information and Declarations

### Data Availability

The following information was supplied regarding data availability: The raw measurements are available in the Supplementary File.
