# Supplementary material for: Clinical characteristics of the severe acute respiratory syndrome coronavirus 2 omicron variant compared with the delta variant: a retrospective case-control study of 318 outpatients from a single sight institute in Japan
Source: PeerJ. 2022 Aug 2;10:e13762. doi: 10.7717/peerj.13762 (PMC9354737; doi:10.7717/peerj.13762)
Supplement: Table S1 [file peerj-10-13762-s001.docx]

|  | **Omicron (n =19)** | **Delta (n =40)** |
| --- | --- | --- |
| Nasal cannulas | 12 (63.2%) | 31 (77.5%) |
| Face masks | 7 (36.8%) | 4 (10.0%) |
| High flow nasal cannula | 0 (0%) | 2 (12.5%) |
